# Medical Items and News of Interest to the Physician

**Published:** 1883-01

**Authors:** 


### Medical Items and News,

### For the Use of Physicians.

CULLED FROM THE STANDARD MEDICAL JOURNALS OF THE WORLD.

NOTE.—These formulæ are intended only for the regular practitioner of medicine, and should be employed only by him, or under his immediate supervision.

**Chilblain Liniment.**

Sulphate of copper. pure, 6 grs.
Eau de cologne.
Distilled water, aa. ½ fl. oz.

Dissolve. Apply twice a day with a camel's hair brush.

**Neuralgia of the Stomach.**

℞
Bi-carb. potassa, ℨ j.
Acid hydrocyanic dil.
Sol. sulph. morphia, aa gtt. xxiv.
Aqua camphor, ℥ vj.

Sig. Teaspoonful as required.—*Louisville Med. News.*

**Acute Rheumatism.**

℞

Sodii salicylatis, ℥ ss.

Glycerinæ, ℥ ss.

Spts. lavandulæ co., ℥ jss.

Liq. ammon. acetatis, q. s. ad ℥ viij.

M. Sig. Take a tablespoonful every three hours.

**Phthisis and Catarrh Cough.**

℞

Mist. ammoniaci, ℥ vj.

Sodæ bicarb, ℨ ss.

Tinct. camph. co., ℨ ii.

Tinct. hyoscyam, ℨ i.

Vin. ipecac. ℨ ij.

Dose. ℨ j. sæpe urgent. tuss.

**Olive Oil in Diseases of the Chest.**

Dr. Parker draws attention to the preference which Prof. Gieth, of Munich, gives to olive oil as a local application in those diseases of the chest in which counter irritation is unnecessary. Prof. Gieth uses a double told of common cotton cloth large enough to encircle the body; the cloth is thoroughly saturated with warm olive oil. This application is said to be very agreeable to the patient, whilst it softens the skin, and is absorbed to a greater or less extent, while at the same time it retains a steady amount of heat.—*Boston Med. and Surg. Journal.*

**Absorbent Powders.**

℞
Powdered maize, ℥ iv.
Oxide of zinc, ℥ i.
Orris powder, ℨ iv.
Essential oil of almonds, gtt. iii.

M. Sig. Dust over surface.—*Medical Gazette*

**Chronic Bronchitis.**

℞

Ammon carb., ℥ j.

Spts. etheris nit.

Syr. scillæ, āā ℥ ss.

Tr. Camph. co., ℨ iij.

Infus. senegæ ad., ℥ viij.

M. Sig. Tablespoonful every four hours. This is especially valuable in the chronic bronchitis in old persons.—*Canada Lancet.*

**Cystits.**

℞

Bals. copaibæ, ℨ iv.

Acid benzoici, ℈ iv,

Gum arabici, ℨ ij.

Sacch., ℨ ij.

Ol. gualtheriæ, gtt. xx.

Aq. camph. q. s. ad., ℥ viij.

M. Sig. Teaspoonful every two hours.—*Medical Gazette.*

**Constipation.**

Dr. J. C. Frye, gives us the following, which he recommends very highly in cases of chronic constipation:

℞

Fl. ex. cascara segrada, ℥ ij.

Tr. belladonnæ, ℨ ij.

Tr. nucis vom., ℨ ij.

Acid nitro-muriat., dil., ℥ ss.

Syrupi, ℥ ij.

Aquæ, ℥ iij.

Dose, a teaspoonful night and morning.—*The Medical Summary.*

**Anti-Asthmatic Mixture.**

Dr. Huchard, of the Hospital Tenon, employs the following, especially when the symptoms of bronchial catarrh are added to the attacks of asthma:

℞

Distilled water, 10 ounces.

Iodide of potassium, 2½ drachms.

Tincture of lobelia, 2½ drachms.

Tinct. polygala, 2½ drachms.

Aqueous extract of opium, 1½ grains.

A tablespoonful to be taken night and morning.

**Bleeding Hemorrhoids.**

℞

Pulv. aluminis, ʒ ii.

Pulv. camphoræ.

Pulv. opii, aa. ʒ i.

Unguent, ℥ i.

M. Sig. Ointment.—*Bartholow.*

**Nocturnal Incontinence of Urine.**

℞

Ex. ural uvæ fl., ʒ xij.

Tr. ferri chloridi, ʒ ij.

Tr. nucis vomicæ, ʒ j.

M. Sig. Teaspoonful in cold water at 3 and 9 p. m. In stubborn cases give at 11 a. m., 3 and 9 p. m., until better, then at 3 and 9 p. m.—*Medical Brief.*

**Dyspepsia.**

℞

Diastase, grs. x.
Pepsin.
Ex. of gentian.
Tartaric acid.
Powdered rhubarb, āā grs. L.
Gentian, q. s.

M. Divide into three grain pills. Dose, two or three pills during meals or before.—*Druggists' Circular.*

**Cystitis.**

Prof. S. W. Gross, of Philadelphia, recommends the following compound, from which he claims "the most excellent results:"

℞

Bal. copaiba, ʒ iv.

Ac. benzoici, ℈ iv

Gummi arabici, ʒ ij.

Sacchari, ʒ ij.

Ol. gaultheriæ, gtt. xx.

Aqua camphoræ, q. s., ad, ℥ viii.

M. S. Teaspoonful every two hours.

**Acid Eructations.**

The following lozengers are superior to the officinal bismuth lozengers:

℞

Bismuth subnitrat, grs. dccxx.

Magnesiæ carbonat, ℥ ij.

Calcis carbon, præcipitat, ℥ iij.

Sodæ bicarbonat, ʒ mdccc.

Sacchari albi ℥ xiv.

Acaciæ gummi, grs. ccvx.

Mucilag. acaciæ, ℥ ij.

Divide into 360 lozengers, and dry them with a moderate heat. From one to six lozengers may be taken at a dose.—*Ibid.*

**Antipyretic.**

Niemeyer strongly recommends the following in pneumonia and other inflammatory fevers:

℞

Sulph. quiniæ, grs. v.

Tinct. digitalis, ♏ xv.

Phos. acid. dil., ♏ xv. M.

Sig. To be taken properly diluted once in four or six hours.—*Am. Med. Journal.*

**Amyl Nitrite as a Remedy for Ague.**

Dr. Saunders, of Indore, India, gives nitrite of amyl mixed with an equal amount of oil of Coriander, to render it less volatile and mask its taste. Four drops of this are put on a piece of lint, and from it is freely inhaled by a patient in the cold stage. Following the flushing of the face which occurs, perspiration takes place, and the cold stage is at an end.—*New Remedies.*

**Chorea.**

Dr. J. H. Carstens, in the *Detroit Clinic*, reports having used the following prescription with success in chorea in a boy eleven years old:

℞

Propylaminæ, ʒ j.

Aquæ menthæ piperitæ, ℥ jss.

Syrupi simplicis, ℥ ss.

M. Teaspoonful three times a day.

**Intermittents.**

Dr. Reed recommends the following formula in the treatment of intermittents:

℞

Quinidiæ sulph., grs. xxx.

Oleo-Resinæ capsici, grs. iij.

Morphiæ sulph., gr. j.

Syrupi, q. s. M

M. Ft. pill, No. x. Sig. One every three hours.

He says that if a cholagogue cathartic be given at the start, and a full opiate (preferably a hypodermic injection of morphia) an hour before the expected paroxysm, this combination has been uniformly successful in his hands, even in most obstinate cases of intermittents.

In cases of malarial cachexia he finds that many recover under the influence of sea air without any medication. When the latter is necessary he has seen excellent results from small doses of nux vomica combined with the sixteenth of a grain of podophyllin, or one-fourth of a grain of blue pill, and repeat every four or six hours.—*Southern Medical Record.*

**Dysmenorrhœa.**

Dr. L. L. Leeds, of Lincoln, Ill., writes us that he has found the tincture of pulsatilla, given in half-drachm doses thrice daily during the interval, to be a most excellent remedy in this affection.—*Med. and Surg. Reporter.*

---

**Tonic in Uterine Complaints.**

℞

Dextro-quiniæ, ʒ j.
Acid hydrochlorici, q. s.
Ferri sulphatis, grs. x.
Strychniæ sulph., grs. j.
Aquæ q. s. ft., ℥ iv.

M. et.

Sig. Teaspoonful three times daily, after eating.—*Review of Med. Phar.*

---

**Facial Erysipelas.**

Dr. V. Netzetzky, of Asiatic Russia, describes a treatment of erysipelas successfully practiced by native barbers. It consists in superficial vertical scarifications made by a razor over the whole diseased surface. After the bleeding spontaneously stops the affected parts are moistened with an aqueous solution of opium (gr. ii. ad ʒ i.), and covered with a layer of cotton wool.—*London Medical Record.*

---

**Diphtheria.**

Mr. T. D. Harris adds another to the already long list of specifics for local application in this disease. In the *Lancet*, February, 1882, page 306, he states that a solution of boracic acid, two drachms to the ounce of a mixture of equal parts of glycerine and water, freely applied to the diseased parts, is a most certain and speedy cure.—R. Neale, M. D., in *London Med. Record.*

---

**Cockle-Burr in Retention of Urine.**

Dr. H. C. Bernard states in the *Therapeutic Gazette* that a case of retention of urine in a little boy, whom he was treating for typhoid fever, was promptly relieved, before a suitable catheter could be procured, by means of an infusion of the common cockle-burr (*xanthium strumarium*) prepared and administered by the patient's grandmother. In all his cases of the same character since, Dr. B. has given cockle-burr tea, and always with such success that he has never had to use an instrument for their relief. He uses fifteen or twenty fresh ripe burrs to a teacup of hot water; a tablespoonful every half hour. A fluid extract of the drug is prepared.

**Chloride of Potassium.**

Prof. Wertheim remarks the mistake that is often made by physicians in using the chlor*ate* instead of the chlor*ide* of potassium as a local application in sore mouths and throats. While the chlor*ate* may in concentrated solution prove very harmful, the other salt is an excellent remedy when used as a gargle.—*Wien. Med. Blatt.*

---

**Sweating.**

In the Michigan *Medical News*, Dr. Currie says that for over thirty years he has used the following prescription, without a single failure, in sweats from whatever cause. Alcohol, Oj, sulphate of quinine, ʒ j. Wet a small sponge with it and bathe the body and limbs, a small surface at a time, care being taken not to expose the body.

---

**Cultivating the Bacillus of Syphilis.**

M. Martineau reports to the Academy de Medicine, that he has cultivated the bacillus of syphilitic chancre, after the method of Pasteur, and has inoculated a pig with the cultivated germs. There resulted a sore and swelled glands, with bacilli temporarily in the blood. The animal recovered in two months. Fluid from its sore did not affect two other pigs inoculated.

---

**Metallic Foreign Bodies in the Eye.**

Dr. Rodriguez reports in the *Revista de Ciercias Medicus*, a very interesting and novel method for removing metallic foreign bodies from the eye by means of an eye wash, composed of ingredients capable of forming a chemical union with the foreign body and transforming the same into a soluble salt, which is washed from the eye by the application. He instances a case of a blacksmith, who, while forging a piece of iron, received a small splinter in his left eye, which remained there incrusted in spite of all attempts at its removal. This wash was then employed, and with extremely satisfactory results:

℞

Rose water, 90 grms.
Iodine, .05 grm.
Iodide of potassium, .05 grm.

Mix. The particle of metal was transformed by this collyrium into a soluble iodide of iron, and all traces of the foreign body disappeared. The cornea regained its normal condition, and the vision remained unaffected.—*Therapeutic Gazette.*

**Beef Tea.**

Virchow, the eminent German physiologist, says that beef tea, which is vulgarly supposed to contain nearly all the valuable elements of the meat, is only of value as a condiment. If drank while warm it is of about the same value as tea or coffee, but contains less heat or flesh-producing substances than wine or beer. It is of high value as a nerve stimulant, and through this property it produces the impression of being itself strengthening.—*The Druggist.*

**Ulcers.**

Dr. Wade, in *Detroit Clinic*, says: In the treatment of ulcers, the cause, if possible, being otherwise removed, I have found no other application to equal the following:

Take of—

Iodoform, powdered, grs. xxx.
Sub-nitrate of bismuth, grs. lx.
Chloral hydrate, grs. xv.
Glycerine, ℥ ij.
Oil of rose geraneum, gtts. x.
Water to make, ℥ iij.

Mix and write. Shake and apply.

**Tincture Ferri Perchloridi.**

Dr. Reed states that a very elegant preparation of the drug may be made by the simple addition to it of a little alkaline citrate. For every drachm of the tincture add half a drachm of potassium citrate. The result is a liquid of a beautiful green color, quite free from the peculiar roughness of the iron. For a tablespoonful dose, containing 10 minims, the perscription may be written thus:

℞

Tinct. ferri perchlor., ʒ ij.

Potass. citratis, ʒ j.

Syrup limonis, ℥ iss.

Aquæ, ad ℥ vi.

—*Canada Med. and Surg. Journal.*

**Ergot in Obstetrics.**

Dr. Williams, of Baltimore, uses ergot in moderate doses, to prevent abortion, at any time during the first three months of pregnancy. In hemorrhage he uses it hypodermically as the only efficient method in his hands. In severe post-partum hemorrhage absorption is so slow and difficult, if not impossible, that he thinks it useless to give it by the mouth. He therefore uses the fluid extract hypodermically, regarding that preparation as more certain in its effects than ergotin, and not liable to produce abscess.—*Trans. Med. and Chirurg. Fac. of Maryland.*

**Chronic Bronchitis.**

℞

Tinct. benzoin co., ʒ ii.

Pulv. tragacanth, ʒ ss.

Aquæ cinnamomi, ℥ iij.

When expectoration is viscid and difficult, the following may be taken:

℞

Vin. ipecac, ♏. v—xv.

Scillæ, ʒ ss.

Oxym scillæ, ♏. v—x.

Decoc. senegæ, ʒ ii—iv.

**Typhoid Fever.**

Writing in the *Pacific Medical and Surgical Journal*, Dr. Oatman announces that iodide of potassium is "as much a specific as quinine is in intermittent fever." His exact plan is this:

An adult with uncomplicated typhoid fever may take five grains of iodide of potassium every three hours in a little sweetened water. Also every three hours one dessertspoonful of the following recipe, viz.:

℞

Ol. terebinth.

Tinct. anisi, aa f ʒ j.

Vitel. ovi, No. ij.

Sacchari, ʒ ij.

Aqæ puræ, ad. ℥ ij.

Ft. emulsio.

M. Sig. This emulsion may be taken between the doses of the iodide.—*Michigan Medical News.*

**Obstinate Vomiting.**

In the course of an article on this subject, in the Boston *Medical and Surgical Journal*, Dr. S. G. Webber says: Often the best method of treating this complication is to give the stomach rest. Sometimes only a large amount of food taken at one time excites vomiting: then it is sufficient to resort to frequent feeding, giving a very small quantity each time, a mouthful or a spoonful every fifteen or thirty minutes; thus the stomach never contains a large mass of food requiring considerable muscular exertion to roll it about, and by its weight or bulk exciting the reflex irritability of the nerve centers. Many times, however, this is not enough; the stomach requires more complete rest, and the best treatment is to withhold all food and medicine; sometimes a few hours' rest is enough, again it requires two or three days; then it will be necessary to use nutrient enemata. Where there has been much vomiting, thirst may be very annoying to the patient; small lumps of ice held in the mouth will relieve this, and generally do not cause vomiting. After the stomach has had sufficient rest, it is best to commence feeding by the mouth, with caution, giving a little frequently. Milk and lime water, equal parts, a teaspoonful every half-hour should be first tried; if well borne the amount can be increased gradually. It is a mistake to increase the quantity too rapidly. *Medical and Surgical Reporter.*

**Asthma.**

℞

Tinct. lobeliæ, ℥ v.

Ammonii iodidi, ʒ ij.

Ammonii bromidi, ʒ iij.

Syr. tolutani, ℥ iij.

M. Teaspoonful every one, two, three or four hours. This gives relief in a few minutes, and sometimes the relief is permanent.—*Fothergill.*

**Quinine and Solution of Beef Enema.**

Take one tablespoonful of brandy, five grains of sulphate of quinine, one teaspoonful of glycerine, two teaspoonfuls of cream, and from four to eight ounces of restorative soup. Mix. This enema may be administered every six or eight hours. Where the rectum is very irritable, or it is necessary to relieve pain, from fifteen to twenty minims of the liquid extract of opium may be advantageously added.—*Med. Gazette.*

**Sycosis.**

According to M. Bouchut (*Jour. de Connoissances Med.*), sycosis may sometimes be cured without epilation by means of applications made morning and evening with an unguent, of which creasote is the active element. He recommends the following formula:

℞

Creasot., ♏ xx ad f ʒ ss.

Zinci oxid., ʒ iss.

Ung. simpl. benzoat., ℥ j.

M. After each application of the ointment the affected parts should be covered with oiled silk.—*Med. and Surg. Reporter.*

**Frontal Headache.**

Dr. Haley says (*Australian Medical Journal* of Aug. 15th, 1881) that, as a rule, a dull, heavy headache, situated over the brows and accompanied by languor, chilliness, and a feeling of general discomfort, with distaste for food, which sometimes approaches to nausea, can be completely removed, in about ten minutes, by a two-grain dose of iodide of potassium dissolved in half a wineglassful of water, this being sipped so that the whole quantity may be consumed in about ten minutes.—*Glasgow Med. Journal*

**Hoarseness.**

Borax has been employed with advantage in cases of hoarseness and aphonia occurring suddenly from the action of cold. The remedy is recommended to singers and orators whose voices suddenly become lost, but which by these means can be recovered almost instantly. A little piece of borax, the size of a pea, is to be slowly disolved in the mouth ten minutes before singing or speaking. The remedy provokes an abundant secretion of saliva, which moistens the mouth and throat. This local action of the borax should be aided by an equal dose of potassium, taken in warm solution before going to bed.—*LaFrance Medicale.*

**Diphtheria.**

Dr. Otto Seifert has used the following as a topical remedy in diphtheria:

Chinoline, 1 gram. (gr. xv.)
Distilled water, 500 gram. (O. i.)
Alcohol, 50 gram. (℥ i.). M.
Oil of peppermint, gtt. ij.

He uses it as a gargle and also applies by means of a brush or swab a solution of equal parts of water and alcohol with five parts of pure chinoline in solution. He thinks that it loosens the membrane in from twelve to twenty-four hours, and that the glandular swellings subside and the temperature is reduced more quickly than under other treatment.

**Abortion.**

Dr. Bell Morrelton reports (*France Medicale*) several cases in which the extract of butternut (juglans cinerea) seemed really efficacious in preventing abortion.

He employs the following mixture:

℞

Ext. hyoscyam., ʒ j.

Ext. juglans cinerea, ʒ j.

Ol. sassafras, ʒ ss.

Sodæ bicarb., ℥ ss.

Syr. simplicis, ℥ vj.

M. A teaspoonful of this mixture may be administered three times daily through the entire period of pregnancy, after the threatened abortion.

The same physician has also employed this remedy in scrofulous affections and as an injection in leucorrhœa.

**Abortive Treatment of Sties.**

As a means of "backing" a sty, Dr. J. P. McGee, of Tennessee, states that the practitioner can use to advantage the following treatment:

Fl. ext. of belladonna, 3 drops.
Water, 2 ounces.

Sig. A teaspoonful every hour.

At the same time he may give calcium sulphide, $\frac{1}{8}$ or $\frac{1}{10}$ gr. every hour, for five or six doses, then every three; although the belladonna is often sufficient alone. Remember, this is sufficient only in the very early stage of the affection—within the first six or twelve hours. He will find it "back" at least three of the five.

---

**Lemonade Iron.**

The effect of prescribing disagreeable medicines for some classes of patients, and especially the roving class which is often met with in the hospital practice, is well known. Some times an artifice in the shape of a *pleasant* medicine is useful in securing the return of a patient, whom it is desired to see again. The following formula for a "lemonade iron" prescribed in a case of this kind was recently mentioned in a clinical lecture by Professor Goodell, of Pennsylvania University:

℞

Tincturæ ferri chloridi, ʒ ij.

Acidi phosphorici diluti, ʒ vi.

Spiritus limonis, ʒ ij.

Syrupi, ad ℥ vi.

M. Sig. A dessertspoonful, in water, after meals.—*The Practitioner.*

---

**Hypodermic use of Quinine.**

I have been informed that the medical profession of Memphis have derived very unsatisfactory results from the administration of quinine hypodermically.

I have been so fortunate as to happen upon a formula given by Prof. Loomis, of New York, in his lectures on fevers, which has given me perfect satisfaction, both in the short time in which I cinchonize my patients, and the freedom from subsequent ulcerations. The formula is as follows:

℞
Quinia bisulph, grs. l.
Carbol. acid, gtt. v.
Sulph. acid, gtt. ii.
Aqua pura, ℥ i. M.

One heyfroder syringeful equals from 3 to 4 grs. of the sulphate given "diam natura."

During the present summer season I have been battling with pernicious fevers in a variety of forms, both intermittent and remittent. In the intermittent it has proven most efficacious. When the attack is of an algidus form, hemorrhagic, or when the congestion is located in the stomach and bowels, adding one-fourth gr. morph. with the first administration in adults, I have been fully satisfied no ulcer is produced, and there is but little inconvenience felt, though there may be some soreness for a few days at the point of injection.—Dr. B. W. Walton in *Miss. Val. Med. Monthly.*

---

**Constipation.**

Where constipation is due to torpor of the muscular layer of the intestine, combined with defective secretion of the mucous membrane, Dr. Bartholow uses either one of these formulæ:

℞
Tinct. nucis vomicæ.
Tinct. belladonnæ.
Tinct. physostigmæ, aa. f. ʒ ij.

Dose—Thirty drops in water, morning and evening, or

℞
Ex. physostigmæ.
Ex. belladonnæ.
Ex. nucis vomicæ, aa. gr. v.
M. et ft. in pil. No. x.

Dose—One pill at bedtime.—*Med. Gazette.*

---

**Hydrocele and Serous Cysts.**

Dr. Levis states that he has been experimenting with a view of determining what substance may best secure the obliteration of the secreting surface, and the adhesion of the walls of the cyst with the most certainty and the greatest freedom from suffering and danger. Having selected carbolic acid as an agent which would provoke simply a plastic inflammation, he injected one drachm of the deliquesced crystals into the sac of a large hydrocele. The new procedure was entirely painless. A sense of numbness alone was experienced, and no inconvenience was felt until, on the next day, the desired inflammatory process developed. A nine-years' hospital and private experience leads the author to believe that this method is the most satisfactory for the object. For the purpose of injection crystalized carbolic acid is maintained in a liquified state by a 5 or 10 per cent. solution of either water or glycerine; the crystals are to be reduced to the fluid state with no more dilution than may be necessary for this. After tapping, inject with a syringe having a nozzle sufficiently slender and long enough to reach entirely through the canula. He has never been able to detect any general toxic effects upon the system, but believes that the action of strong carbolic acid on surfaces secreting albuminous fluids is to seal them, to shut them off from the system in such a way that absorption can not readily take place. The occluding influence of strong carbolic acid he regards as an important surgical resource in certain cases of compound fracture, destructively lacerated wounds, and ulcerating surfaces where septic infection is inevitable. All forms of serious cysts which are usually subjected to any form of operative treatment, on the principle of producing plastic adhesion of their walls, may be deemed amenable to the treatment indicated.—*Medical News.*

**Eczema.**

The following has been found useful in the hands of M. A. Wilson, M. D.;

Relative to general eczema, acute and subacute, with debility, anorexia, constipation, etc., I wish to give a formula which I have found very useful; in one case especially the effect was remarkable, the eruption subsiding within a few days, no local treatment being employed. A few months afterward the same prescription relieved the same patient quickly from a second attack.

℞

Magnes, sulph., ℥ x.

Ferri sulph., gr. xv.

Acid. sulph., dil., fl ʒ ij.

Tinct. calumbæ, fl ℥ j.

Tinct. capsici, ♏ xl.

Syr. zingiberis, fl ℥ j.

Aq. menth. pip., q. s. ad fl ℥ vj.

M. Sig. A teaspoonful in a half-glass of water two or three times a day.

Quinine and nux vomica can also be added, if necessary. Dieuretics, especially potas. acet., in half-drachm doses, and plenty of water, are much employed at the Bellevue Dispensary.

**Cough Medicines.**

We present a few simple recipes for expectorants, useful for winter coughs. The first is particularly useful for young children:

Syrup of squill, 1 fl drachm.
Gum acacia, powdered, ½ drachm.
Ammonium chloride, 8 grains.
Peppermint water enough to make two fluid ounces.

Dose for a child, a teaspoonful every two hours.

Another formula, for adults and older children, consists of

Syrup of ipecac, 2 parts.
Syrup of squill, 4 parts.
Paregoric, 1 part.

Dose, half to one teaspoonful, repeated as often as necessary.

The following was a favorite prescription of the late Prof. C. A. Lee, of Peekskill:

Syrup of ipecac, 1 ounce.
Syrup of tolu, 1 ounce.
Paregoric, ½ ounce.
Syrup of wild cherry, 1 ounce.

FOR HOARSENESS.

Dr. Eichelberger gives the following, which he says is very good for hoarseness:

Tinct. chloride of iron, 2 drachms.
Glycerine, 4 drachms.
Water, 4 drachms.

Dose, half a teaspoonful *pro re nata.*

**Chronic Eczema.**

The so-called papaine, the partially purified extract obtained from the pawpaw tree, was recommended at the International Congress last year as a solvent of diphtheritic false membrane. I have lately been using it as a local application in old standing cases of chronic eczema, more especially of the palm. As it is used in the West Indies to soften tough meat, I thought it might be of service in cases the chief feature of which is thickening and hypertrophy.

It would occupy too much space to discuss the method of action here; it will be sufficient to say that it is supposed to digest certain substances by means of a special ferment. Roughly speaking, it may be said to resemble pancreatine. I have had such marked success with it, that I recommend it to the profession as a remedy worthy of trial in cases which often resist all the ordinary modes of treatment.

The following brief notes of a case recently treated may be of interest. Mrs. R., was sent to me by Dr. J. E. Pollock, last June. She was suffering from severe chronic eczema of both palms, which competely resisted the external use of tar, and mercury ointments of various kinds and strengths, as well as a six months' course of arsenic, and was only slightly relieved by prolonged soaking. On April 18th, I ordered 12 grains of papaine and 5 grains of powdered borax, in two drachms of distilled water, to be painted on the parts twice daily. When seen on May 9th, the hard horny masses of heaped-up epidermis, which were present on April 18th, had entirely disappeared from the skin, covering the heads of the metacarpal bones, leaving its texture quite normal. The fissures of the central portion of the palms had quite healed, and the cuticle was softened and pliable, though still showing signs of roughness.

The patient stated that the application produced only a slight pricking and tingling sensation. No internal remedies were given. The borax was added with the view of checking fermentation, and supplying the alkali required in order to obtain the full action of the drug. In some other cases of chronic eczema and psoriasis, the same benefit has followed in as short a time as in the case above mentioned.—*British Medical Journal.*

---

**Scrofulous Ulcers of the Cornea.**

This is a form of keratitis which often comes under the care of the general practitioner, and as injudicious treatment may lead to the formation of opaque cicatrices, the following recommendations regarding treatment, made by M. Dehenne, in Le Progres Medical, may prove of service:

1. Instill daily into the eye four or five drops of the following collyrium:

℞

Atropiæ sulphat (neutral), gr. j.

Aquæ destillat., f ℨ iiss.

M.

2. Abstain from every form of metallic collyrium, which often leave indelible marks, veritable metallic leucomata. Do not use any collyrium containing eserine, for affections of the cornea are frequently accompanied by iritis, and there would be danger of formation of posterior synechiæ.

3. Each evening insert between the eyelids, with a small camel's hair pencil, a portion, about as large as a pea, of the following unguent:

℞
Hydrarg., oxid. flav., gr. xv.
Ung. petrolei, ℥ ss.
M.

4. Apply four times daily over the eye compresses soaked in warm chamomile tea.

5. Administer a tablespoonful of cod-liver oil every morning.—*Med. and Surg. Reporter.*

---

**Chorea.**

At the end of a paper on chorea, based upon an experience of one hundred cases (*British Medical Journal*, Volume II, 1881, page 145), Dr. William Strange speaks of the treatment, saying that the changes must be rung on the so-called nervine tonics, varying them according to the temperament of the child or to the collateral symptoms accompanying the choreic movements. If pallor, palpitations, and loss of weight exist, iron or arsenic, or both, will be necessary. If, on the contrary, the vascular system be sufficiently full and the motile element prevail, then the bromides with ammonia or the succus conii, will be of most avail. Frequently whatever the condition of the vascular system and of the general nutrition, no good arrives until we have succeeded, by sedatives, in calming the excessive mobility of the nervous system. In these cases, Dr. Strange has used the ice-bag to the spine and the ether spray to the nape of the neck, but not with much success. Direct calmatives—digitalis, belladonna, cannabis indica, with the bromides—answer the best.

The nervous symptoms once quieted, iron or arsenic may now be given, and carried to a somewhat high degree. Some have recommended large doses of arsenic, ten to fifteen minims of Fowler's solution; but Dr. Strange has seldom found that the stomach will tolerate these large doses, and has contented himself with much smaller ones, in combination with iron or zinc.

But, whatever the remedy selected, it will be necessary to continue its administration until it has produced its special physiological effect. Especially is this necessary with the neurotic sedatives. Children bear large doses of belladonna and conium; and Dr. Strange has never found this class of remedies do much good until their full physiological effects (consistent with safety) have been produced.

Dr. Strange used some years ago, to treat all his cases of chorea with wine alone, the port wine of the hospital, merely clearing out the primæ viæ, to make sure that trouble was not caused by entozoa or depraved alvine secretions. The amount given was three to six ounces daily, and all the cases got well. After suspending this treatment for some years, he has recently recommenced it with good results.—*The Canada Medical Record.*

**Rheumatism.**

As a remedy in rheumatism manaca has proven specially efficacious in my hands, but more notably in a case of inflammatory rheumatism.

Mrs. ——, æt. 35, had been bed-ridden for three months with inflammatory rheumatism. All the various remedies, such as guaiacum, salicylic acid, potassium bromide, hypodermic injections of morphia, etc., had been used, previous to my being called in, without success.

I immediately resorted to the manaca as in the following prescription:

℞
Fluid ext. manaca, gtt. 40.
Syrup simp., q. s. ft. ℥ j.
M. ft. mistura.

Sig. Teaspoonful every three hours until the peculiar effects of the drug are produced.

These peculiar effects are a sense of tightness and fullness about the head with profuse perspiration.

The first prescription produced none of the special symptoms, so I renewed the medicine, doubling the dose of manaca, thus:

℞
Fluid extract manaca, gtt. 80.
Syrup simp., q. s. ft. ℥ j.
M. ft. mistura.

Sig. Teaspoonful every three hours until the peculiar effects of the drug are produced.

This prescription sufficed, the special symptoms of the drug were produced, and the patient recovered within a week.

The manaca, after the special symptoms had been produced, was continued in ten drop doses three times a day for a week.

It seemed as if all symptoms of rheumatism disappeared directly after the peculiar effects of the drug manifested themselves by head symptoms, etc., and recovery was almost immediate, but for reasons of safety I continued to administer it in ten drop doses three times a day, for a week, after which time all symptoms of her peculiar disease had disappeared.

I have always found in administering the drug, manaca, that it was necessary to have the peculiar effects of the drug, as exhibited by head symptoms, etc., show before any curative effect was experienced.—Dr. Pepper, Connersville, Ind., in *Therapeutic Gazette.*

---

**Epilepsy.**

M. Ball, the present professor of mental diseases at the Paris School of Medicine, considers that the drugs most used in epilepsy prove much more efficacious when taken in combination with each other, than when one of them is administered singly. The alkaline bromides particularly the bromide of ammonium, with belladonna and oxide of zinc, form the basis of treatment.

The following solution may be given, in tablespoonful doses:

℞

Ammonii bromid., ʒ iiss.

Sodii bromid., ʒ iiss.

Aqua destil., f ℥ x. M.

At the commencement of treatment four tablespoonfuls of this solution may be taken during the day, and the dose increased to eight or ten tablespoonfuls if no appreciable effect is noticed after a few days.

Belladonna and oxide of zinc are administered in pill form, as follows:

℞
Ext. belladonnæ, gr. xv.
Zinci oxid., gr. xv. M.
Ft. pil. xl.

Sig. One pill may be taken in the morning, another in the evening, at first; then the dose may be increased to four pills per diem.

If any degree of plethora exists the drastic purgatives should be resorted to, and in some cases benefit is obtained from a general bleeding, or the application of leeches to the temples or behind the ears.

M. Ball gives the following formula for drastic pills:

℞
Aloes, gr. xv.
Scammon, resin, gr. viiss.
Jalapæ resin, gr. viiss.
Calomel, gr. viiss.
Saponis medic., q. s. M.
Ft. pil. xxiv.

Sig. These pills are to be taken once a week, three in the morning and as many more about noonday,

What is of importance to notice is the immediate beneficial effect of this combined treatment; this is sometimes remarked on the second day.

This method, like all other forms of treatment of epilepsy, should be continued for a long period, and should not be suddenly stopped; the doses should be progressively and slowly diminished when it is considered safe to lay aside the treatment.—*Med. and Surgical Reporter.*

